# Development and validation of the SickKids Enterprise-wide Data in Azure Repository (SEDAR)

**DOI:** 10.1016/j.heliyon.2023.e21586

**Published:** 2023-11-02

**Authors:** Lin Lawrence Guo, Maryann Calligan, Emily Vettese, Sadie Cook, George Gagnidze, Oscar Han, Jiro Inoue, Joshua Lemmon, Johnson Li, Medhat Roshdi, Bohdan Sadovy, Steven Wallace, Lillian Sung

**Affiliations:** a Program in Child Health Evaluative Sciences, The Hospital for Sick Children, Toronto, Canada; b Information Management Technology, The Hospital for Sick Children, Toronto, Canada; c Division of Haematology/Oncology, The Hospital for Sick Children, Toronto, Canada

**Keywords:** Electronic health records, Microsoft Azure, Schema, Validation, OMOP-CDM

## Abstract

**Objectives:**

To describe the processes developed by The Hospital for Sick Children (SickKids) to enable utilization of electronic health record (EHR) data by creating sequentially transformed schemas for use across multiple user types.

**Methods:**

We used Microsoft Azure as the cloud service provider and named this effort the SickKids Enterprise-wide Data in Azure Repository (SEDAR). Epic Clarity data from on-premises was copied to a virtual network in Microsoft Azure. Three sequential schemas were developed. The Filtered Schema added a filter to retain only SickKids and valid patients. The Curated Schema created a data structure that was easier to navigate and query. Each table contained a logical unit such as patients, hospital encounters or laboratory tests. Data validation of randomly sampled observations in the Curated Schema was performed. The SK-OMOP Schema was designed to facilitate research and machine learning. Two individuals mapped medical elements to standard Observational Medical Outcomes Partnership (OMOP) concepts.

**Results:**

A copy of Clarity data was transferred to Microsoft Azure and updated each night using log shipping. The Filtered Schema and Curated Schema were implemented as stored procedures and executed each night with incremental updates or full loads. Data validation required up to 16 iterations for each Curated Schema table. OMOP concept mapping achieved at least 80 % coverage for each SK-OMOP table.

**Conclusions:**

We described our experience in creating three sequential schemas to address different EHR data access requirements. Future work should consider replicating this approach at other institutions to determine whether approaches are generalizable.

## 1 Introduction

In healthcare, the data generated by the electronic health records (EHR) are a rich resource that can be used to address diverse institutional needs. Data are used for administrative purposes and operational reporting. For example, data on emergency department encounters, ambulatory clinic visits and admission rates as well as census data are critical for resource planning and administrative reporting. EHR data may be central to quality improvement projects, particularly related to resource utilization such as laboratory tests or prescribing of medication. Finally, EHR data are key to facilitating research endeavors. EHR data may be used to identify patient cohorts, to create datasets for observational research, to be an efficient adjunctive data source for laboratory based, observational or interventional trials and may also be the foundation for machine learning. Many institutions have begun to develop and implement patient-focused predictive machine learning models, often using EHR data as the primary data source. These models can improve clinical outcomes such as sepsis [1], clinical deterioration [2], acute care visits [3] and mortality [4].

While EHR data are required for multiple purposes, there are well-known barriers to utilization, including difficulties with data access and data curation [5,6]. An EHR may have one or more non-relational or relational databases. These databases may be highly complex, making it difficult to query them correctly and efficiently. Direct access to these databases is typically restricted because of the potential to impact existing workflows and difficulty in managing privacy risks when provisioning access. Finally, institutions typically create parallel streams to address administrative and research needs, essentially duplicating the required work.

Given the wide-ranging needs for EHR data and the challenges associated with effective utilization, we reasoned that it would be efficient to conceptualize, develop and deploy a curation pipeline in a single repository to meet needs across the institution. We also reasoned that different users may require access to different levels of curated data, and that an efficient approach might be to create sequential schemas with increasing degrees of curation. Accomplishment of these activities using a cloud-based approach would create a scalable solution and could leverage cloud-based tools such as those for resourcing and provisioning compute. It also allows for tailored expenses since resources are only implemented or increased when required and thus, the institution would only pay for services or compute capacity required. Consequently, the objective was to describe the processes developed by The Hospital for Sick Children (SickKids) to enable utilization of EHR data by creating sequentially transformed schemas for use across multiple user types.

## 2 Materials and methods

### 2.1 Literature review

To describe what is known about similar EHR data management approaches, we conducted a systematic review with the assistance of a library scientist and searched for articles indexed from database inception to August 31, 2023. We searched MEDLINE including Epub ahead of print, in-process and other non-indexed citations, and Embase. We included articles describing a data curation process focused on EHR data. We excluded studies focused on the following: a single disease, condition or test; natural language processing; data curation for the purpose of a specific machine learning use case; pipelines involving non-EHR data; and conference abstracts. Appendix 1 shows the full search strategy.

One reviewer (LS) screened titles and abstracts and identified potentially relevant articles for review at full text. Eligible studies were narratively described.

### 2.2 Establishment of the environment

We used Microsoft Azure as the cloud service provider and named this effort the SickKids Enterprise-wide Data in Azure Repository (SEDAR).

### 2.3 Data source and establishment of the transformation pipeline

At the time this work was conducted, the SickKids EHR was Epic. The Epic data model contains three databases populated with EHR data: Chronicles, Clarity, and Caboodle. Chronicles stores the real-time data in a hierarchical, non-relational format and is not suitable for general reporting. Clarity is a relational database derived from Chronicles according to an extract-transformation-load (ETL) schedule set by the institution, with the most common frequency being daily. Clarity is the main reporting database. Finally, Caboodle is a star-schema relational database that further transforms and normalizes data from Clarity. We chose to start with Clarity for our data curation pipeline. To avoid negative impacts on existing Clarity-based hospital activities, we created a copy of Clarity and transferred the copy to Microsoft Azure. Once the copy of Clarity was in Azure, we created three sequential schemas, each with a distinct purpose.

First, SickKids shares its Epic instance with another tertiary care pediatric institution named the Children's Hospital of Eastern Ontario (CHEO), located in Ottawa, Canada. Some patients may be seen at both institutions, but most patients would only be seen at one of the two institutions. We were mandated to remove CHEO data in the downstream schemas, and thus, the first step in the pipeline removed CHEO patients, encounters and activities as well as invalid patients for a subset of Clarity tables. Apart from applying the filters, the resulting tables maintained the structure of the original Clarity tables. We named this schema the Filtered Schema and it represented selected Clarity tables in which CHEO patients, encounters and activities and invalid patients were removed. This schema might be useful for those with broader access to the Clarity data who required tables that had already been filtered to include only SickKids data.

Second, Clarity data are highly normalized, and SickKids Clarity currently includes approximately 18,000 tables. The location of clinically or operationally relevant data is often situational, and related items can be separated across multiple source tables. This structure makes Clarity difficult to understand, navigate and query. The second schema was considered the main transformation and was named the Curated Schema. The purpose of this schema was to facilitate understanding and utilization of the data. Each table contained a logical unit such as patients, hospital encounters, laboratory tests or medication administrations as examples. Each table included key dates and times such as datetime ordered, datetime obtained and datetime resulted for laboratory tests, and datetime ordered and datetime administered for medication administrations. The purpose of this schema was to facilitate most uses that require Protected Health Information (PHI). Such uses were anticipated to include operational reporting and quality improvement projects.

The third schema aimed to facilitate multicenter observational research and machine learning by adopting the Observational Medical Outcomes Partnership Common Data Model (OMOP CDM) [7]. The OMOP CDM allows systematic analysis across different data sources by using a common structure and vocabulary. We used OMOP CDM version 5.4 without modification and we named this schema the SK-OMOP Schema. Development of this schema required three distinct tasks, namely concept mapping, establishing an ETL from the Curated Schema and data de-identification. The goal of concept mapping is to represent each medical entity with a common concept id across all OMOP CDM databases. Concept mapping leveraged existing medical coding where available such as institutional designation using International Classification of Diseases (ICD) 9 and ICD 10 codes, and Logical Observation Identifiers Names and Codes (LOINC). To achieve concept mapping for remaining entities, data from the Curated Schema were loaded into Usagi, a software tool that facilitates the mapping between source data and concepts in OMOP vocabularies [8]. Two reviewers conducted the mapping, one of whom had clinical expertise (EV and LS). The goal was to map at least of 80 % of rows in each SK-OMOP table. The ETL used the concept maps as lookup tables and mapped categorical and text data from the Curated Schema into the SK-OMOP Schema based on the OMOP CDM specifications. De-identification steps included randomly generating surrogate numbers for identifiers such as patient medical record number, encounter id and order id, applying the shift and truncate method [9] to obscure date information for clinical events and patient characteristics, suppressing non-numerical and non-mapped source data and suppressing the notes table in the initial iteration.

### 2.4 Data validation

Because of the complexity of Epic databases and the transformations between them, we anticipated errors in a naïve transformation from the Filtered Schema to the Curated Schema. Our goal was for SEDAR to reflect the data viewed by clinicians and administrators using Epic Hyperspace (Epic's front end graphical user interface application), to ensure that downstream reports matched what users would expect. Errors were anticipated to encompass incorrect information, duplications and omissions. For example, Epic Hyperspace would display a single timestamp to reflect the time a specific laboratory test was ordered. If the time ordered for that laboratory test in Curated Schema was incorrect (since timestamps related to the laboratory test order are obtainable from multiple tables and columns depending on the workflow and type of order), this would be considered an error. Similarly, if the laboratory test was shown as two separate rows (duplicated) or missing in Curated Schema, these also would be considered errors. Thus, we validated each table in the Curated Schema against Epic Hyperspace for accuracy and completeness. Accuracy was assessed on randomly sampled activities (for example, 100 laboratory tests or medication administrations) to examine whether each attribute (such as datetime ordered, datetime obtained, datetime resulted, value and unit for laboratory tests) matched values observed in Epic Hyperspace. Completeness was assessed on all activities for randomly sampled encounters and patients to examine whether there were missing or duplicated records. Completeness was defined as all observed units in Epic Hyperspace (such as laboratory tests or visits) being present in Curated Schema. Errors identified were not subjective. For example, the time a laboratory test was ordered would be clearly available in Epic Hyperspace. Similarly, duplications or omissions in Curated Schema would be clear. Thus, inter-rater reliability was not evaluated. If errors were identified, these were corrected by understanding the nature of the error and by modifying the data transformation process. The updated table was re-evaluated in the next iteration of randomly sampled observations. Iterations were repeated until no errors were found.

Validation required three distinct types of personnel. Data engineers (LLG, JI and JL) created the ETLs. Clinical research associates (MC and SC) compared each data element in the Curated Schema tables for randomly sampled observations against their view in Epic Hyperspace and indicated correct and incorrect data. To resolve errors, an Epic analyst (LS) viewed incorrect data in Chronicles to identify the Chronicles and Clarity fields corresponding to the correct data. The process of resolving errors entailed a weekly meeting between all validation team members (data engineers, clinical research associates and Epic analyst).

### 2.5 Ethical considerations

The SickKids Research Ethics Board (REB) approved the use of SEDAR for the purpose of research (REB number: 1000074527).

## 3 Results

### 3.1 Literature review

The search strategy identified 306 references. After duplicates were removed, we screened 240 potentially relevant articles, of which 14 were retrieved for full text evaluation. Nine met eligibility criteria and were included in narrative description [[10], [11], [12], [13], [14], [15], [16], [17], [18]]. Almost all were focused on creating a research data warehouse. None involved data use across multiple purposes (such as administrative, operational and research purposes) and none used data validation to iteratively refine the ETL process.

### 3.2 Establishment of the environment

The initial environment used a virtual private network tunnel to transfer the Clarity data to Azure and used a single virtual network to host the Clarity data and downstream schemas. To make the process scalable, we subsequently enabled Microsoft Azure ExpressRoute to transfer Clarity to Azure rather than relying on a virtual private network tunnel. We also later segregated the network and data by creating separate network and data hubs, each with their own virtual networks.

### 3.3 Data source and establishment of the transformation pipeline

For the initial load of Clarity, data were copied to an Azure file share and restored to a virtual machine. After the initial load, log shipping was used to update the copy of Clarity and the data were restored each night, completing by 4 a.m. Log shipping is a process whereby transaction log backups from a primary instance (Clarity) are copied and restored to a secondary instance (Clarity copy in SEDAR), thus allowing Clarity copy to reflect the updated data in Clarity.

**Fig. 1:**
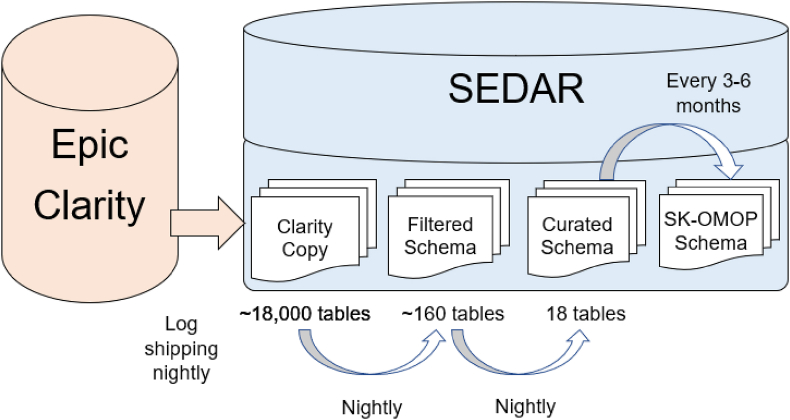
Overview of SEDAR transformations.

We then developed the ETLs using stored procedures resulting in the Filtered, Curated and SK-OMOP Schemas (Fig. 1). Each of the three schemas included an approach to update the data. The Filtered Schema was updated daily via incremental and full loads. Incremental loads utilized change tracking tables in Clarity, which track incremental changes to the corresponding Clarity tables that support row update tracking. Incremental loads extracted only newly added or updated rows in Clarity since the last ETL. Full loads were performed on Clarity tables that do not support row update tracking including dimension tables that contain attribute values for categorical data. Filtering of CHEO patients, encounters and activities were performed during the transformation stage for both incremental and full loads.

The Curated Schema included the following 18 tables: patient, non-hospital encounter, hospital encounter, admission-discharge-transfer (ADT), laboratory, microbiology, pathology, blood bank, medication administration, patient-controlled analgesia bolus, prescription, clinical procedure, imaging, flowsheet, note, diagnosis and diagnosis summary. Diagnosis summary was an alternate view of the diagnosis table. Updates to the Curated Schema tables were performed daily via incremental loads that considered only changes in the Filtered Schema since the last ETL.

A problematic table was flowsheet, as there were approximately 30,000 flowsheet measurement ids in SickKids’ Clarity. As it was not feasible to validate all of these, we started with a subset of clinically relevant items such as respiratory rate, heart rate, blood pressure and temperature as examples. A request for a new item, namely vomiting, was received during the development process and thus, the corresponding flowsheet measurement ids were located and validated. The general approach will be to add and validate new flowsheet measurement ids as they are requested by users.

For concept mapping required to create the SK-OMOP Schema, we were able to use existing institutionally assigned ICD-9 and ICD-10 codes for diagnosis, LOINC for a subset of laboratory tests and Drug Product Database codes for a subset of medications. For medications without an assigned Drug Product Database code, we also used the RxNorm web service (https://www.nlm.nih.gov/research/umls/rxnorm/index.html) to obtain RxNorm concept unique identifiers (RxCUI) using the generic drug name, and subsequently mapped the RxCUIs to OMOP medication concepts. Across all SK-OMOP tables, concept mapping for >80 % of rows was achieved (Appendix 2). Tables were updated using full loads from the Curated Schema with a plan to update every 3–6 months.

In addition to daily ETLs, we implemented a reconciliation process that is executed once a week to identify and reconcile Filtered Schema tables for which there is a discrepancy in the number of rows from the source (Clarity). Changes as a result of the reconciliation process were then applied to the Curated Schema and SK-OMOP Schema.

### 3.4 Data validation

**Fig. 2:**
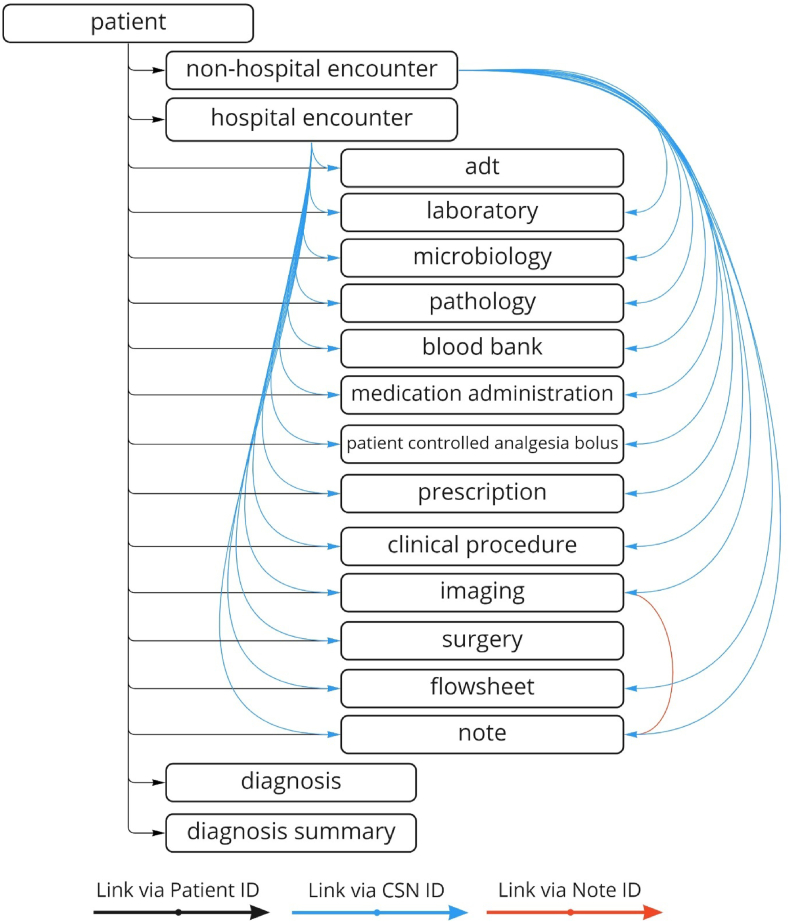
Tables included in SEDAR curated Schema.

Table 1 and Fig. 2 show the tables in the Curated Schema. Table 1 shows the number of iterations required to complete validation of each table, with microbiology and flowsheet requiring the largest number of iterations. The number of minutes required to validate each iteration ranged from 30 to 120 min. Fig. 2 shows how the different Curated Schema tables are linked. Overall, the ETL creation and validation process of the Curated Schema took 13 months. Table 2 shows common challenges encountered during data validation.

**Table 1:** SEDAR curated Schema tables and iterations required to validate data.

Table	Iterations Required
Patient	8
Non-hospital Encounter	3
Hospital Encounter	7
ADT	7
Laboratory	13
Microbiology	16
Pathology	5
Blood bank	4
Medication Administration	15
Patient-controlled Analgesia Bolus	7
Prescription	2
Clinical Procedure	3
Imaging	4
Surgery	4
Flowsheet	16
Note	9
Diagnosis	14
Diagnosis Summary	NA^a^

a Diagnosis summary is an alternate view of the diagnosis table.

**Table 2:** Common challenges encountered during data validation of curated Schema.

Challenge with Source Data	Examples	Solutions
Ambiguous source column definition or name	Difficult to associate the time of a specific clinical event to a specific column in Clarity	Access to Chronicles (source of Clarity and Hyperspace) to identify the Clarity column(s) for a specific clinical event
Workflows determine which columns contain the correct information	Order time exists in different columns for different types of orders such as regular and pended orders	Need to understand the workflow to create a conditional query
One clinical entity can have data with different levels of detail and structure	Details for admission, transfer and discharge events exist for hospital encounters but not non-hospital encounters Medication administration, prescription and patient-controlled analgesia bolus derived from different source tables, each with different structure	Created separate tables (hospital encounter and non-hospital encounter) to accommodate level of detail Created separate tables (medication administration, prescription and patient-controlled analgesia bolus) to accommodate structure
Size and diversity of flowsheets	Approximately 30,000 flowsheet measurement ids	Started with a small and tractable set of common flowsheet items for understanding and validation, then expand as requirements are identified
Source data not observed in front end user interface	Some non-hospital encounter types not viewable in Hyperspace such as history, laboratory requisition and wait list	Included in SEDAR because may be associated with required data such as laboratory tests or flowsheet data Noted the potential for discrepancy with Hyperspace in the user manual

### 3.5 Early utilization of the data

For the first six months following completion of Curated Schema, only two analysts were able to access the environment for the purpose of creating hospital-based reports for decision support. During this period, 20 projects were completed and included both single reports as well as Power BI-based dashboards. In addition, SK-OMOP was released for research purposes.

## 4 Discussion

We provided an overview of our approach to enable utilization of EHR data through copying data to a cloud computing solution and then creating three sequential schemas to address multiple institutional needs. Data validation was central to our process. Our intention in sharing this experience is to facilitate similar work at other organizations with comparable goals.

One of the important aspects of our work is that we envision curation and validation efforts to be centralized and to benefit users across the institution including administrators, clinicians and researchers. While others have developed research repositories focused on data in EHRs, fewer have developed a data pipeline to meet institutional data needs more broadly. Further, while some descriptions exist [19], a more detailed report may help other institutions make design choices in developing their own cloud-based or on-premises EHR data repository.

The described work began with Epic Clarity data as an important data source required by multiple stakeholders. However, we envision incorporating other data sources to create a linkable repository. Other planned data sources will include real time data, waveforms, imaging, genetic data and legacy data.

In placing this work in relation to the wider literature, the systematic review identified the lack of previous efforts focused both on data curation across multiple purposes as well as using data validation as an iterative approach to refine the ETL. This review highlights the uniqueness of our effort and emphasizes the importance of the description of our data curation approach. While we could only describe early utilization of the system, this utilization was encouraging, including the successful release of the OMOP data for research purposes.

The strengths of this manuscript include a description of the end-to-end processes institutions will need to consider if planning similar transformations of their EHR data. Validation of data in the Curated Schema and dual review of OMOP concept mapping were other strengths. The inclusion of a Filtered Schema is another strength as it may be applicable to many situations where one EHR instance is shared between multiple institutions. However, the report is limited in that we are unable to share the developed ETLs because of privacy and security considerations. Mechanisms to share experiences, successes and challenges among multiple institutions may be useful to address this challenge. Another limitation is that we did not discuss considerations related to various cloud database services as these may depend on institutional needs, the cloud service provider and the mechanism by which the source data is moved.

## 5 Conclusions

We described our experience in creating three sequential schemas to address different EHR data access requirements. Future work should consider replicating this approach at other institutions to determine whether approaches are generalizable. Important limitations include inability to share developed ETLs broadly and focus on a single cloud database service. Enabling multiple institutions to leverage data in their EHR to conduct multi-center research, for example, using the OMOP CDM, has the potential to accelerate progress globally.

## Funding

This work was partially supported by the 10.13039/501100017559Garron Family Cancer Centre.

## Data availability statement

There is no database associated with this manuscript. The code cannot be made publicly available because of the proprietary nature of institutional electronic health records data and the potential risk to patient privacy. Please see Discussion section that addresses this issue.

## Authors contributions

LG, MC, JI, EV, and LS drafted the manuscript. All authors provided important scientific contributions, revised and approved the manuscript, and agree to be accountable to all aspects of the work.

## Declaration of competing interest

The authors declare that they have no known competing financial interests or personal relationships that could have appeared to influence the work reported in this paper.

## Figures and Tables

**Table:** 

#	Searches	Results
1	medical records systems, computerized/or electronic health records/or health information exchange/	46696
2	((electronic or computer*) adj2 (record* or chart*)).ti,ab,kf.	68617
3	or/1-2	92967
4	Data Curation/or (data adj2 curat*).ti,ab,kf.	2424
5	3 and 4	151

**Table:** 

#	Searches	Results
1	electronic health record/or electronic health record certification/or “meaningful use criteria"/	40504
2	electronic medical record system/	2579
3	((electronic or computer*) adj2 (record* or chart*)).ti,ab,kf.	128223
4	or/1-3	139302
5	(data adj2 curat*).ti,ab,kf.	2227
6	4 and 5	155

**Appendix 2:** OMOP Concept Mapping Rate by OMOP Table^b^

OMOP Table	Percept Mapped Standard OMOP Concepts
condition_occurrence	85.4 %
device_exposure^a^	0.0 %
drug_exposure	
drug_concept_id	81.3 %
route_concept_id	99.0 %
measurement	
measurement_concept_id	97.4 %
unit_concept_id	100.0 %
value_as_concept_id	100.0 %
note	85.3 %
observation	
observation_concept_id	100.0 %
observation_type_concept_id	100.0 %
person	
ethnicity_concept_id^a^	0.0 %
gender_concept_id	99.8 %
race_concept_id^a^	0.0 %
procedure_occurrence	94.2 %
specimen	87.9 %
visit_detail	100.0 %
visit_occurrence	95.0 %

a We did not have device in Curated Schema and we did not have race or ethnicity in SickKids Clarity.

b Percent mapped for each table related to concept_id except where noted otherwise.

## Appendix 1 Search Strategies

Ovid MEDLINE: Epub Ahead of Print, In-Process & Other Non-Indexed Citations, Ovid MEDLINE® Daily and Ovid MEDLINE® <1946-Present>

## Embase

Embase Classic + Embase <1947 to 2023 August 30>
